# Transition Metal Chalcogenides: Perspectives on Their Applications for Nitrate Reduction

**DOI:** 10.1002/open.70270

**Published:** 2026-08-17

**Authors:** Martins O. Omorogie

**Affiliations:** ^1^ Chair of Urban Water Systems Engineering School of Engineering and Design Technical University of Munich Garching Germany; ^2^ Department of Chemical Sciences Faculty of Natural Sciences Redeemer's University Ede Nigeria

**Keywords:** heteroatoms‐rich vacancies, hydrogen evolution reaction (HER), NH_3_ yield, nitrogen reduction reaction (NRR), transition metal chalcogenides

## Abstract

This review paper highlights the research on thrives for nitrate reduction by transition metal chalcogenides (TMCs) to produce ammonia. The exposure of humans to nitrate in the environment via public drinking water supplies is on the increase due to the elevated usage of inorganic fertilizers and animal manure in agricultural lands in various regions of the world. Many regions of the world has already set a permissible limit for nitrate in drinking water so as to prevent methemoglobinemia in children, birth defects, thyroid dysfunction and disease, various cancers, and cyanosis that culminates into asphyxia. Also, serious health disorders may occur due to ingestion of excess nitrate, such as cardiovascular disorder, genetic mutation, and diabetes. Recently, TMCs have attracted the keen interest of the scientists/researchers due to their unique tunable properties such as heterojunction formation with noble metals, morphological engineering through various synthesis techniques, elemental doping, and tunable edge active site and surface defects that induce active sites transformation at the basal planes. Irrespective of these unique surface properties, TMCs have the shortcomings of increasing surface hydrogen binding energy and Gibbs free energy change, which enhances charge‐carrier separation and hydrogen evolution reaction (HER). Future research outlook should focus on the surface engineering of TMCs in terms of minimizing the formation of heterojunction, which will limit proton/electron‐transfer kinetics, shifting the chemical equilibrium of HER through catalyst engineering that will mitigate the adsorption of hydrogen species, regulating the crystal phase, blocking the HER active sites, and introducing strain effects that will suppress HER and boost nitrogen reduction reaction (NRR). These strategies will suppress HER and increase the efficiency of NRR.

## Graphical abstract

This review paper reports the syntheses, properties, and applications of TMCs for electrochemical reduction of nitrate to ammonia. Various studies revealed that the conversion of nitrate to ammonia is principally the electrocatalytic hydrogenation of nitrate and the rate and yield of ammonia is competitively challenged by hydrogen evolution reaction.

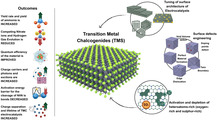

## 1 Introduction

Global agricultural revolution in recent years has caused the leaching of nitrate from the soil into surface and groundwater. Nitrate leachate from intensive agricultural activities in both developed and developing countries of the world is on the increase due to the usage of fertilizers with very high nitrogen contents that are applied in soils in which various food crops, such as fruits, vegetables, cereals, legumes, tubers, etc*.,* are planted [1, 2, 3, 4, 5]. The excessive application of nitrate fertilizers in agricultural farmlands is responsible for non point source nitrate pollution that cascades into the leaching of high concentrations of nitrate into surface water and groundwater especially [1, 2, 3, 4, 5, 6, 7, 8, 9, 10, 11, 12, 13]. Accumulation of nitrate in plants and animals increases its toxicity indices and the rate of its conversion from nitrate and nitrite to value‐added chemicals, such as ammonia [14, 15]. The transformation products/intermediates of nitrates leached from the soil into surface water and groundwater result from the excessive loading of the soil with fertilizers. This has led to an increase in the salinity and acidification of the soil, sharp decrease in the activities of soil microbes and enzymatic activities (such as sulphatase, arylsulphatase, phosphatase, ß‐glucosidase, etc.) [16, 17, 18, 19, 20]. Nitrate‐contaminated drinking water and dietary consumption of nitrate‐contaminated food cause methemoglobinemia (blue baby syndrome); cancers of the digestive tract (enteritis); compromised cardiovascular system functioning; elevated levels of nitric oxide in human blood, irregular rises in blood pressure, leukemia, brain tumors in infants; and heart disease mismanagement [15, 21, 22, 23, 24]. Also, eutrophication of water bodies and algal blooms and greenhouse gas contribute immensely to global warming respectively [25, 26, 27, 28]. The European Commission (EC), United States Environmental Protection Agency (USEPA) and World Health Organisation (WHO) permissible limit of nitrate in drinking water is ≤50 mg.L^−1^ [22, 29, 30]. Also, the WHO and European Food Safety Authority established the daily permissible nitrate intake of ≤3.7 mg.kg^−1^ body weight [31]. To mitigate this pollution problem and enhance environmental sustainability, there is a dire need for nitrate to be reduced into ammonia via, a value‐added chemical that is useful as a viable source for renewable clean energy to meet global energy demand, cogent energy storage medium [32] and an industrial heavy chemical [32, 33, 34]. Nitrate reduction is achieved by reductants (electrons and water) in the presence of protons for conversion into nontoxic valuable chemicals [35, 36, 37, 38]. The world ammonia production is forecast to reach $76.64 billion by 2025 [39, 40].

Transition metal chalcogenides (TMCs), a class of nanomaterials which possess a wide array of physicochemical properties, have demonstrated some unique potential in recent times as facile electrocatalysts. Surface engineering of TMCs due to crystal engineering, surface tuning via atom doping, heterojunction formation, introduction of defects, enhancement of charge carrier separation (slow recombination of charge carriers), tuning of band gap energy, and modification of their active sites have made them promising electrocatalysts for applications in photodetectors, energy storage devices, energy conversion devices, optoelectronics, transistors, spintronics, and secondary batteries [41, 42, 43, 44, 45, 46, 47, 48, 49, 50]. TMCs thin films and nanocrystals have improved electroactive sites for structural flexibility, redox reactions tendency, and electronic properties. These properties have made viable and attractive materials for new electrodes designs in comparison to the existing traditional materials [51]. The surface engineering and tuning of TMCs must reach a compromise of improving nitrogen reduction reaction (NRR), at the same time, reducing hydrogen evolution reaction (HER) via blocking their active sites, slow recombination of charge carriers, effectively manipulate the technical strategies of heteroatom doping and cocatalysts addition that improves the active sites for hydrogen adsorption and reduce the binding energy of these active sites so as to suppress HER. Also, the electronic structures of TMCs can be modulated to change their lattice symmetry and atomic structure [48, 52, 53, 54, 55, 56]. The layer‐by‐layer or island‐like growth of TMCs is determined by the localized super‐saturation of the selective precursor(s). The accumulation of selective precursors in the synthesis route of TMCs induces the formation of patterned crystals that give various regions of vacancy [57].

Also, there are predictions that a myriad of TMC materials are 2D magnets, which require synthesis feasibility and structural and environmental stabilities that need experimental validation [58, 59]. Also, they have been identified as suitable electrodes as a result of their engineered stoichiometric compositions, dynamic crystal structures, elevated charge separation efficiency, rich redox sites, possess the tendency to form heterojunctions with staggered alignment of the energy bandgap and relatively higher electrical conductivity when compared to their transition metal oxide counterparts [60, 61, 62]. In the last decade, TMCs have successfully been used in NRR process for the generation of value‐added chemicals. Generally, the efficiency of the NRR process decreases drastically due to the presence of active sites that support HER. These active sites accelerate HER process by the adsorption of hydrogen ions in rapid successions, and this leads to the blocking and exhaustion of active sites that would have been available for NRR process. Against this backdrop, the quest to strategically develop NRR process that is unhindered by highly competitive HER process is on the rising demand. Hence, the strategic design of TMCs is of utmost cogency in achieving highly successful NRR process that will increase active sites via the introduction of vacancy and dopancy, construction of heterostructure to improve conductivity, effective utilization of off‐symmetry growth and out‐of‐plane growth features, and developing large interlayer space that will promote defect engineering and nanostructure engineering [60, 63, 64].

Nitrate reduction is pertinent to the sustainability of the ecosystem due to the deleterious menace that excessive nitrate can cause to the ecological balance of the entire biota [15, 65]. Conventional technologies such as ion exchange, adsorption, reverse osmosis, electrodialysis, and photocatalysis have been reportedly applied for the removal of nitrate from water. These aforementioned technologies have some drawbacks. They have been found to be ineffective, laborious, nonbenign, nonsustainable and inefficient. In recent times, electrocatalysis has been proven to be a reliable, benign, sustainable, easy, effective, and efficient technology for the reduction of nitrate over a wide array of experimental conditions. Also, the electrocatalytic NRR maximizes catalyst design and optimization, catalyst selectivity, flexibility in possible outcome delivery, ease of integration into groundwater treatment, and energetically favorable bond energy (N—O bond; 204 kJ.mol^−1^ in comparison to N≡N bond; 941 kJ mol^−1^) [34, 38, 66, 67, 68, 69, 70, 71, 72]. Some electrocatalysts have been used for nitrate reduction, which include alloy catalysts, nanofibers, perovskite oxides [73], transition metal (TM) oxides, TM/graphitic carbon nitrides, metal‐free doped carbon, TM‐molecular catalysts, nonnoble metal catalysts, bimetallic catalysts, noble metal catalysts, single‐atom catalysts, and dual‐atom catalysts, among others [15, 74, 75, 76, 77, 78]. This review unveils the progress on the applications of TMCs for nitrate reduction.

## 2 Transition Metal Chalcogenides (TMCs)

**FIGURE 1:**
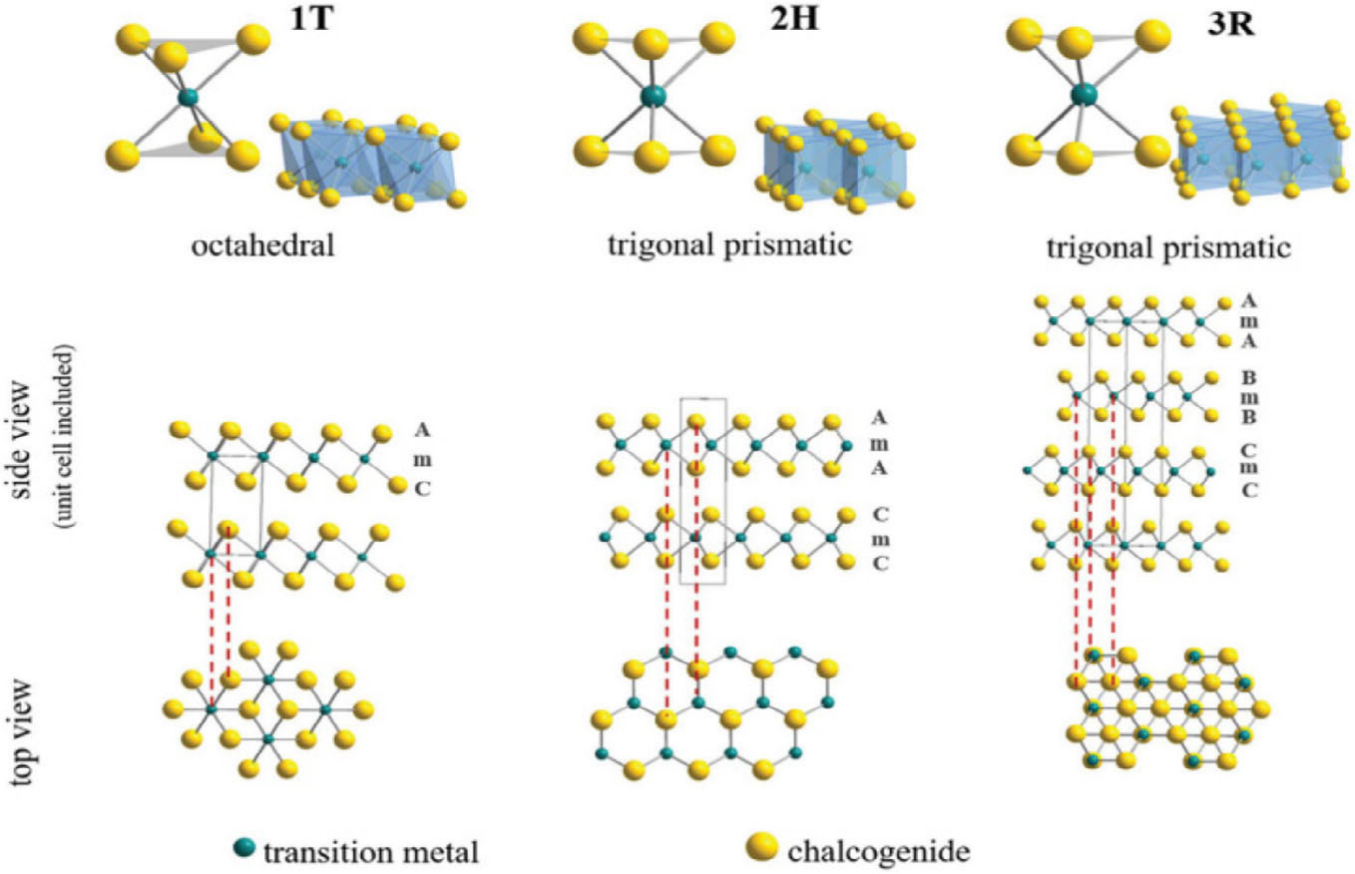
The schematic illustration of TMCs [79]. Reproduced with permission: Copyright 2021, Royal Society of Chemistry.

TMCs are crystal‐based materials that are stable anisotropies comprising groups 3–12 and 16 transition metals, especially sulfides, selenides, and tellurides (Figure 1). TMCs have excellent physicochemical properties such as benignity, bio‐compatibility, cost‐effective, facile synthesis routes, material diversity, tunability, exceptional stoichiometry, distinct and variable energy band gaps, excellent charge separation, low electron–hole recombination, etc.. Moreover, TMCs possess varying morphology and texture, diverse quantum effects, electrochemical active sites, short diffusion pathways, good flexibility, moderate conductivity, among others. These excellent physicochemical properties of TMCs confirm their suitability as excellent candidates for electrocatalysts for nitrate reduction [80, 81, 82, 83, 84, 85]. Their structures consist of at least one chalcogen anion (S_2_, Se_2_, or Te_2_) and at least one cation. TMCs have drawn salient attention due to their aforementioned physicochemical properties [60, 85]. Over the years, Researchers have made significant progress over the application of various synthesis routes, which are hydrothermal, solvothermal, hydrothermal, sol–gel, hot injection, etc*.*, for the synthesis methods for TMCs. The application of TMCs as semiconductors in the visible light region is possible based on their physicochemical properties, positioning them at a vantage position as photoelectrocatalysts for the most effective, efficient, and benign route for nitrate reduction [86, 87, 88, 89, 90]. Hence, it becomes very imperative to develop suitable photoelectrocatalysts for nitrate reduction.

The physicochemical properties of TMCs are evident in their photoactive reaction mechanisms, which are (a) narrow and tunable energy bandgaps, which reduce electron–hole (e^−^–h^+^) recombination to the barest minimum. This tends to reduce charge separation and transfer; (b) efficient charge separation of photo‐generated charge carriers (e^−^–h^+^); (c) high capacity and tunability for light harvesting in the visible region, which is a function of high quantum efficiency; (d) fast migration of photo‐generated charge carriers from valence band (*V*_B_) to conduction band (*C*_B_); (e) tunable growth and surface assemblage into high surface area‐to‐volume ratio, with enhanced reactivity sites, fostering improved the photoactivity; (f) stability under prolonged light exposure, so as to prevent photo‐corrosion [84, 91].

## 3 Synthesis Pathways of TMCs

Some techniques have been reported for the syntheses of TMCs and their modified analogs. Even though the interfacial, structural, and morphological properties determine the photoactivity of TMCs, it is extremely pertinent to design their synthesis routes/pathways. These synthesis routes/pathways such as hydrothermal/solvothermal technique, electrospinning, one‐pot heat‐up technique, hot‐plate technique, etc*.*, are presented as follow.

### 3.1 Hydrothermal/Solvothermal Technique

The principle of the hydrothermal/solvothermal technique lies in the fact that it uses an autoclave method in the presence of solvent/water to drive the synthesis process at elevated temperatures. At elevated temperature, there is a cogent interfacial energy disparity among solvents and the particles of precursors. This technique applies the Teflon‐lined high‐temperature and chemical‐resistant vessel, enclosed in a pressurized stainless steel reactor. The autoclave is heated beyond the saturation temperature of the solvent/water, and this increases the pressure inside the reactor. The amount of pressure maintained within the reactor is a function of types of solvent/water, volume of solvent/water, and temperature of the Teflon‐lined vessel. An unregulated environment will make this synthesis technique to initiate the production of crystals, which generate seeds preceding aggregation. The optimization of stability in such an environment will lead to the formation of spheres and decrease in specific surface area. It should be noted that any modification will enhance adjustment in nucleation and growth direction that promotes surface recrystallization and pore development. This technique promotes the formation of a variety of porous structures for TMCs engineered to specific modifications, even though it is an expensive and high‐power consuming method [92, 93, 94, 95, 96, 97].

**FIGURE 2:**
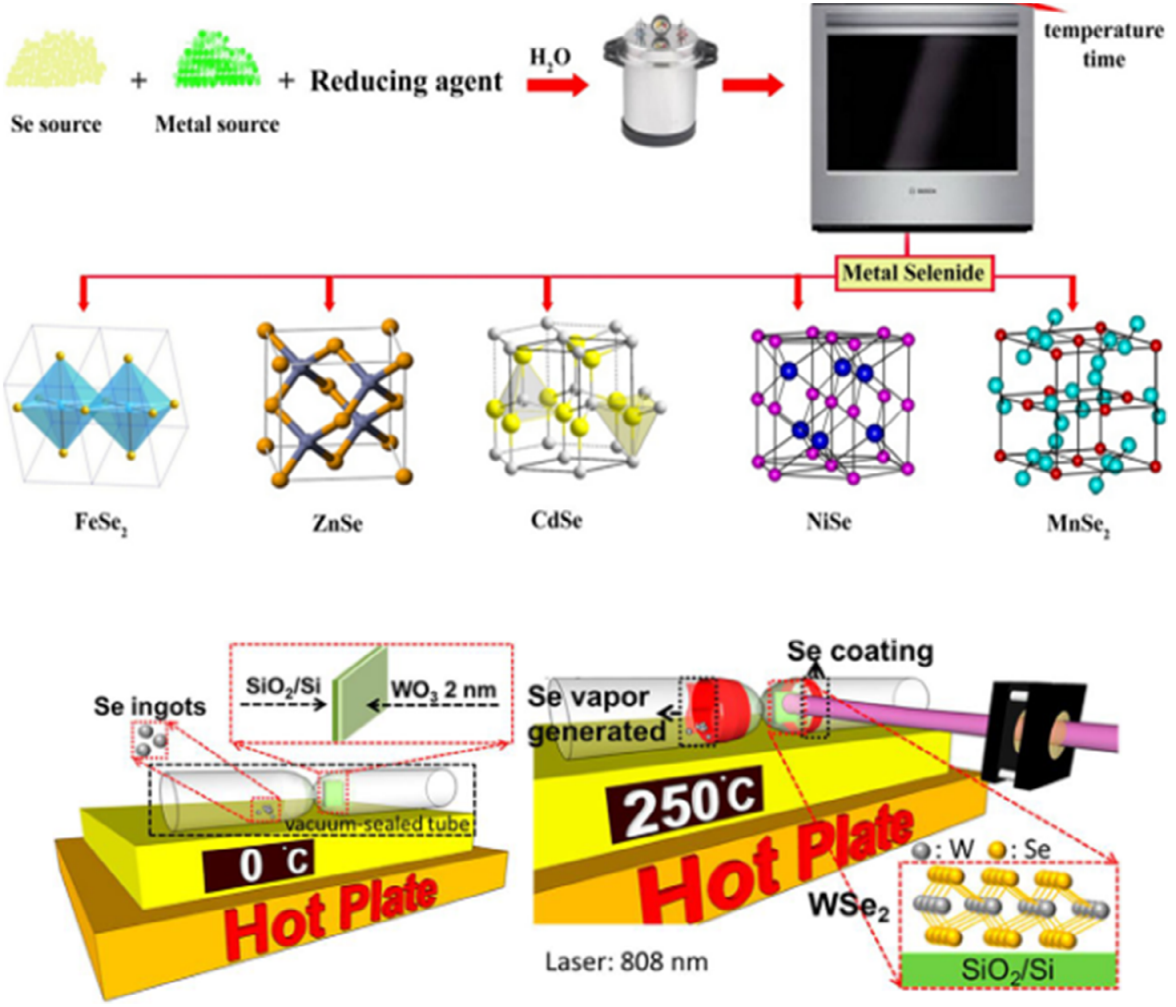
(a) The hydrothermal synthesis pathways for various TMCs [104]. Reproduced with permission: Copyright 2021, Elsevier Publisher, (b) Synthesis of TMCs by hot‐plate technique [105, 106, 107]. Reproduced with permission: Copyright 2015, American Chemical Society and Copyright 2024, Springer Publisher.

Also, the degree of nucleation and crystallinity are strongly determined by other synthesis conditions, such as type of solvent, pH, reaction time, concentration of precursor, reducing agent, oxidizing agent, surfactant, to mention a few [80, 92, 93, 94, 98]. On the contrary, solvothermal technique for the syntheses of TMCs is done at reaction temperatures lower than those of hydrothermal syntheses and results in perfect crystal nucleation and growth [99]. But, recent progress on the synthesis of TMCs has shown that hydrothermal technique has gained continuous attraction. Murugadoss et al. [100] synthesized cobalt–nickel selenide dispersed over graphene nanohybrids; Akyüz et al. [101] synthesized reduced‐Cd_(1−*x*–*y*)_Zn_*x*_Mo_*y*_S, reduced graphene oxide with Cd_(1−*x*–*y*)_Zn_*x*_Ni_*y*_S and reduced graphene oxide Cd_(1*−xy*)_Zn_*x*_Cu_*y*_S decorated with TMCs of Cd, Mo, Ni, and Cu, Liu et al. [102] synthesized four quaternary silver‐based sulfides Ba_2_Ag_2_XS_4_ (X = In, Sn, Ge); and Zhang et al. [103] synthesized MnS nanorods by anodized aluminum oxide. Also, Salavati‐Niasari et al. synthesized various TMCs by hydrothermal technique in their studies (Figure 2a) [108, 109], which are NiSe, NiSe_2_, Ni_3_Se_2_ [110, 111], CdSe [112], FeSe_2_ [113], MnSe_2_ [114], CoSe [115], CuSe, Cu_1.8_Se, CuSe_2_, Cu_3_Se_2_ [116], ZnSe [117] and Cui et al. [99] synthesized Cs_2_CdGeSe_4_, Cs_2_Hg_2_GeSe, and Na_3_RbCu_8_Ge_3_S_12_. Also, the synthesis of other TMCs [118] by hydrothermal technique was used for [Mn_2_SnS_4_(N_2_H_4_)_2_] [119], Cu_2_NiSnS_4_ [120], ZnSe_2_ [121, 122, 123], CdS [124], CdSe [122, 125], Cu_2−*x*_Se [126], Co_9_S_8_ [127], SnSe [128], Bi_2_Se_3_ [129], InS_2_ [130], MSe_2_ (M = Ni, Co, Fe) [131], ME_2_ (M = Ni, Co, Fe, Mo; E = S, Se) [132], CuIn(Se_*x*_S_1−x_)_2_ [133], CuInSe_2_ [134], AgMS_2_ (M = In, Ga) [135] and CuMS_2_ (M = In, Ga) [136].

### 3.2 Electrospinning Technique

Electrospinning is quite a multidimensional and resourceful technique that is used for the synthesis of TMCs, which involves the transfer of selected high voltage current to the syringe tip immersed with suitable viscous precursors [92]. Electrospinning utilizes the principles of electrospray with the high‐voltage electrostatic forces via propel liquid jets. This technique uses nozzle, collector, and high‐voltage generated through alternating current or direct current. The strong electrostatic field counteracts the surface tension of TMC precursors influenced by some physicochemical properties like conductivity, viscocity, and molecular weight and process parameters such as flow rate, voltage, and distance between the nozzle and collector [137, 138]. Electrospinning is quite a multidimensional and resourceful technique that is used for the synthesis of TMCs, which involves the transfer of selected high‐voltage current to the syringe tip immersed with suitable viscous precursors.

Electrospinning is controlled by some parameters, such as environmental, process, and solution parameters. Some of these cogent parameters of electrospinning are density of the precursor (*ρ*) (g.cm^−3^), feed rate (*Q*) (mL.h^−1^), electric field intensity (*E*) (V.m^−1^), electric current (*I*) (*A*), diameter (*R*) (cm), and collecting distance (*D*) (cm). These parameters are expressed by the Spivak model analysis, which is given as [139, 140, 141]

(1) $$R=\left(\frac{{\left(\rho Q^{3}\right)}^{1/4}}{{\left(2IED\pi \right)}^{\frac{1}{4}}}\right)$$

Maximal optimization of these parameters is critical and beneficial toward engineering the diameter and structure of electrospun fibers. The applied voltage operates in preponderance to the amount of charge carrier, which controls the production of the electrospun fibers below or equivalent to critical value. In pragmatic terms, applied voltage explores a high‐level electric field intensity that delivers better stretching of the precursor(s), favors the fabrication of electrospun fibers with tiny diameters, and optimizes the control of fiber size, provided the feed rate of the precursor is constant over time [141]. Some TMCs synthesized by electrospinning are Ni_3_S_2_/Ni [142], Fe_7_S_8_/ N‐doped carbon fibers [143], FeS/C [144], carbon nanofibres of CoSe_2_ [145, 146], MoSe_2_ [146], ZnSe/CoSe_2_ [147] and MnSe [148].

### 3.3 One‐Pot Heat‐Up Technique

The principles of one‐pot heat‐up technique lie in the production of monodispersed TMCs, which are reproducible on a large‐scale synthetic pathway. This synthetic technique is paramount for decreasing variations in batch‐to‐batch processes when considered for commercialization. This technique reduces concentration gradients and temperature in reaction mixture. The application of this technique in large scale results in the production of polydispersed TMCs which are obtained from the nucleation of TMCs under various chemical and physical parameters and does not require size selection to decrease size distribution of TMCs in large‐scale processes. The striking merit of this technique is that reactants are mixed at low temperature and then heated up to high temperature [149].

The one‐pot‐heat‐up technique is a holistic approach, energy efficient, time efficient, and simplistic/facile technique which utilizes the precursors of TMCs in a mono‐step reaction synthesis to produce the desirable TMCs [80, 92]. This technique favors controllable syntheses of TMCs for highly acquiescent large‐scale production [80, 92, 150]. Literature shows the following TMCs synthesized by one‐pot‐heat‐up technique, which are Co_9_S_8_/rGO/Ni_3_S_2_ composite [151], NiCo_2_Se_4_ and NiCo_2_Se_4_/rGO [152], Cu‐doped Ni_3_S_2_ [153, 154], MnS/MoS_2_/C [155], WS_2_‐ReS_2_ [156], Cu_4_SnS_4_ [157], TiSe_2_, NbSe_2_ [158], ZrSe_2_, HfSe_2_, VSe_2_, NbSe_2_, and TaSe_2_ [159], CdY (Y = Te, Se, and S) [160], ZnY and PbY (Y = Te, Se, and S) [83, 161, 162, 163, 164, 165 *]* *.*

### 3.4 Hot‐Plate Technique

This principle of the hot‐plate technique involves the dissolution of precursors in water, preceding heating in hot plate that initializes the chemical reaction. Thereafter, the reaction mixture is heated and calcined at high temperature, resulting in the formation of TMCs. Some parameters such as reactants’ ratio, calcination temperature, and time are responsible for desirable morphology control. This morphology is achieved via growth in the shape and size of monocrystalline nanostructure of TMCs as gradual temperature increase is achieved [92, 166, 167].

This technique involves the application of a steel or metal plate that is heated to a significantly high temperature, so as to enhance the oxidation of TMC precursors and promote the nucleation of TMCs on the hot‐plate surface. The nucleation temperature is maintained at low level, leading to the formation of TMC vapors from their respective precursors [168]. The hot‐plate technique is strictly controlled by the choice of TMC precursor(s), time, temperature, capping agents, and molar ratios. These conditions/parameters help to maximize the efficiency of this technique, resulting in well‐structured TMCs [92, 166, 169]. This technique has been used to synthesize CdS‐WS_2_ [170]. This led to the formation of TMCs with regular interconnectivity, uniformity, and homogeneity in single‐crystal nanostructures, shape, and size. It is noteworthy to mention that the spraying of the TMC precursors on the hot plate must be meticulously and steadily done in order to keep the temperature constant and prevent the slow formation of TMCs due to over‐cooling effect. To minimize the impact of this effect on the synthesis of TMCs, the precursors should be sprayed on the hot plate intermittently at an interval of 5 min. This step will lower the over‐cooling effect significantly or completely eliminate it (Figure 2b) [171]. This technique has been used to synthesize CdTe, ZnTe, In_2_S_3_, Bi_2_S_3_, Sb_2_S_3_, Sn_2_S_3_, CuInS_2_, CuInSe_2_, Cd_*x*_Zn_1−*x*_S, CdSb_2_S_4_, Cu_2*x*_SnS_4_ [105, 172], NiS, SnS_2_, and Cu_2_S [105, 173, 174].

### 3.5 Hard‐Templating Technique

The principle of the templating technique is effective for the synthesis of hollow TMC nanostructures after the removal of the templates by selective etching or chemical reaction. However, hard template employs strategic synthetic three‐stage route of utilized template with desirable microstructure. The encapsulation of the hard template produces a target layer or intermediate that helps in the specific removal of its hard inner core via chemical etching or high‐temperature calcination. A hard template of high quality produces good monodispersity that is a prerequisite for the assemblage of materials with complex matrices of various chemical compositions. The hollow structure of TMCs is advantageous for effective transmission of electrons and ions and enhanced electrochemistry [175, 176, 177].

**FIGURE 3:**
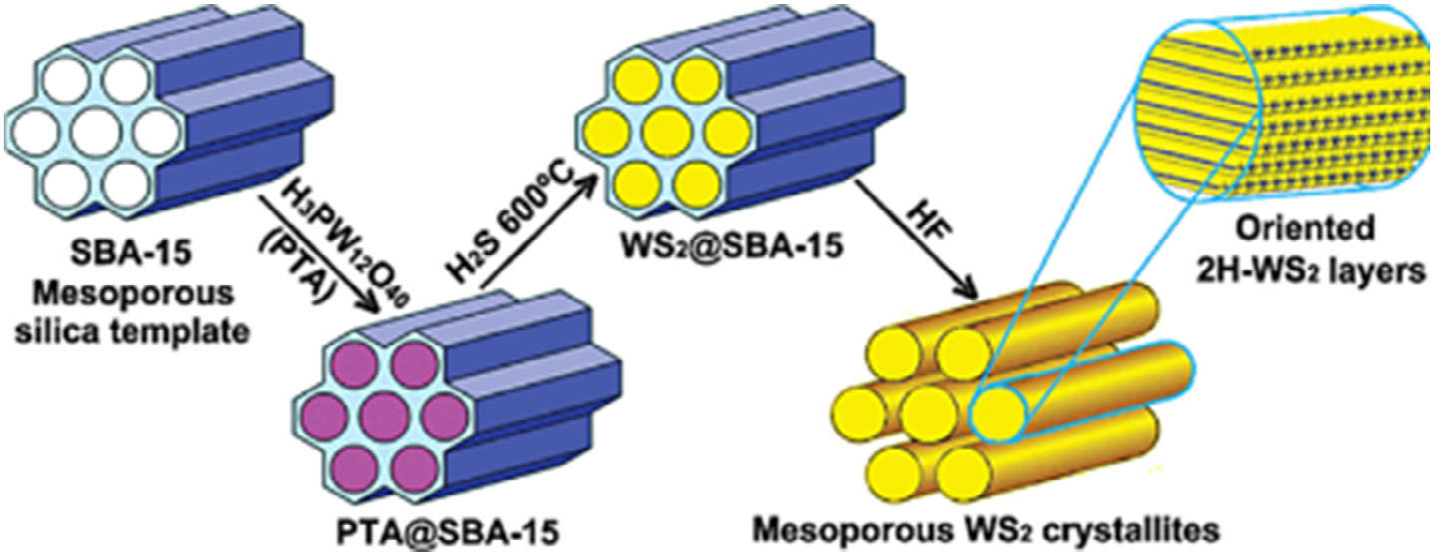
Hard‐template synthesis by SBA‐15 for 2D hexagonal well structured porous WS_2_‐phosphotungstic acid (PTA) (H_3_PW_12_O_40_) [178]. Reproduced with permission: Copyright 2007, American Chemical Society.

Over the years, hard‐templating technique has been used for the syntheses of TMCs. This technique does not require the strict control of template concentration, evaporation rate, crystallization rate, condensation rate, organic/inorganic interactions, humidity, temperature, etc*.* (Figure 3). This technique utilizes exotemplates (Korean Institute of Technology‐6 (KIT‐6), Santa Barbara Amorphous (SBA)−15, Fudan University‐5 (FDU‐5) and Mobil Composition of Matter −48 (MCM‐48)) that metamorphose into hard porous solids that are replicas [179, 180, 181, 182, 183, 184, 185, 186].

This technique has successfully been applied for the syntheses of structurally ordered porous materials with high level interconnectivity into which desired TMC precursors are chemically, thermally and hydrothermally incorporated to obtain the engineered phases. The incorporation of the desired TMC precursors into these exotemplates plays self‐supportive role and the rigidification of the final replica mesostructures if done repeatedly. This exotemplate is removed by HF or NaOH after the final mesostructures are solidified or rigidified enough to desirability. Hard‐templating technique has been applied to synthesize well‐ordered KIT‐6 and SBA‐15 two‐dimensional, hexagonal, three‐dimensional and bicontinuous cubic heterostructured ZnSe, ZnS, CdS, CdSe, CdS_*x*_Zn_1−*x*_Se, CdS_*x*_Zn_1−*x*_S), CuS, Ag_2_S, polyoxometalate/Ag_2_S/CdS, NiS_2_, FeS_2_, CoS, WS_2_, WSe_2_, MoS_2_, and MoSe_2_ [178, 187, 188, 189, 190, 191, 192, 193, 194, 195, 196]. Irrespective of the fact that this technique has been known to be efficient for the syntheses of TMCs of intrinsic porosity and crystallinity, it is non facile, laborious, expensive, and non tunable (restricted control over shape, pore size, and occasionally surface area). Hence, these pitfalls in this technique place strong limitations of difficulty in obtaining TMCs with regular porosity and specific pore texture [186].

### 3.6 Exfoliation Technique

The exfoliation technique finds an array of applications for the syntheses of TMCs. It has successfully been explored for the syntheses of synthetic or natural crystalline TMCs of very high quality; of which the transition metals and chalcogens do not undergo chemical interaction. This technique requires the cleavage of bulk crystals on adhesive layers, which are in contact with target substrates that are mechanically pressed (mechanical exfoliation).

Exfoliation comprises a monolayer of TMCs and two outer closely‐packed chalcogenide planes sandwiched within transition metal layer. It is understood that TMCs consist of weak out‐of‐plane van der Waals interactions and strong‐in‐layer bonds. This method facilitates the delamination of TMCs into monolayers through physical and chemical methods. Physical exfoliation uses the micromechanical approach at limited production scale, separation of materials into various geometries with no change in the chemical composition or crystal phase. External forces such as swift expansion of supercritical solutions, shear mixing, and ultrasonication are considered as salient forces that help in the dispersion of microcavities that generate a high‐frequency pressure differential for exfoliation formation [176, 197].

A few layers of TMCs are formed on the target substrate after the adhesive layers have been removed. Mechanical exfoliation leads to low yield, limits scalable synthesis, and reduces lateral dimensions. Also, the syntheses of TMCs could be oxygen‐involving processes of plasma cleaning and heating/cooling of the transition metals and chalcogens. This will enhance the crystals‐substrate adhesion (adhesion‐controlled exfoliation).

The synthesis of single‐to‐few layered TMCs is achieved by liquid‐phase exfoliation, which is essential for a cascade of electrocatalytic applications. For this synthesis route, there is a selective intercalation of nanocrystals from transition metals and chalcogens to form interlayer spaced‐TMCs via surfactant‐assisted exfoliation, intercalation‐induced exfoliation, and solvent‐assisted exfoliation [198, 199, 200, 201, 202, 203, 204, 205, 206]. Noteworthy to mention is that the solvent‐assisted exfoliation is simplistic and efficacious for the production of monolayer and multilayer TMC crystals in colloidal solutions. Some TMCs synthesized by exfoliation techniques are MoS_2_ [207, 208, 209, 210, 211, 212, 213, 214, 215, 216], WSe_2_ [209], WS_2_ [210], TaX_2_ and VX_2_ (*X* = S, Se, Te) [217, 218], TiS_2_, TaS_2_, and ZrS_2_ [216].

### 3.7 Chemical Vapor Deposition (CVD) Technique

CVD is an efficient technique for the synthesis of TMCs that possess sound quality of crystals to release larger‐crystal nucleation with similar thickness. CVD is effective in decreasing high melting point of precursors and nuclear density, as this increase the rate of chemical reaction on the solid templates [97].

CVD is an efficient, effective and facile technique used to synthesize high quality TMCs with excellent crystallinity, engineered morphology, tunable, etc*.*. The chalcogens and transition metal precursors are exposed to the substrate(s) at elevated temperature and pressure, which leads to the deposition of TMCs on it [219, 220, 221, 222], such as MX_2_ (*X* = S, Se, Te) [221, 222, 223, 224, 225, 226]. The modification of TMCs with dopants, alloys and heterostructures will assist in tuning their magnetic and opto‐electronic properties [227, 228, 229, 230, 231, 232, 233, 234]. Also, CVD could achieve uniform sediment deposition on the substrate with limitless macrostructure. This involves the diffusion of gas(es) and deposition on the substrate at normal or low pressure to produce a phase‐structured layer, regulated by different experimental variables. This is applicable for large scale production with relatively low deposition rate and difficulty in achieving local sedimentation [235, 236, 237]. For instance, the intercalation of *V*_1+*X*_S_2_‐V_3_S_5_ [236]. Table 1 shows the morphology engineering, cogent process parameters and pros/cons for the synthesis of different TMCs.

**TABLE 1:** Morphology engineering, significant process parameters, and pros/cons for various synthesis methods for TMCs.

Synthesis technique	Pros	Cons	Morphology engineering	References
Hard templating (mesoporous silica SBA‐15, KIT‐6, colloidal silica as sacrificial scaffolds) Template‐assisted.	• Precise control over pore size, geometry (2D hexagonal, 3D bicontinuous cubic) • High surface area products ideal for catalysis and energy storage • Applicable to CdS, WS_2_, MoS_2_, NiS_2_, CoS_2_, and heterostructures • Reproducible and compositionally versatile	• Multistep process: template preparation → infiltration → template removal • Harsh etching (HF or NaOH) needed to remove silica template • Risk of structural collapse after template removal • Limited to templatable compositions; not easily scaled	MediumOrdered mesoporosity; moderate scale	[176, 186, 238, 239, 240]
Exfoliation (mechanical tape, liquid‐phase sonication/shear mixing, gold‐assisted, Li‐intercalation).	• Mechanical exfoliation yields highest crystal quality, pristine monolayers • Gold‐assisted variant gives large‐area flakes (100 µm) • Liquid‐phase exfoliation (LPE) is low‐cost and gramme‐scalable • No high‐temperature or vacuum equipment required for LPE	• Tape exfoliation: tiny flakes (<10 µm), very low throughput • LPE: polydispersed thickness/size; defects from sonication • Li‐intercalation: phase transition (1T) and restacking issues • Difficult to integrate into wafer‐scale device fabrication	Low–mediumNanosheets; limited lateral size control	[241, 242, 243]
Chemical vapor deposition (CVD) (powder‐CVD, molten‐salt‐assisted CVD, MOCVD, PECVD).	• Controlled layer number and uniform lateral growth on diverse substrates • In situ alloy, heterostructure, and dopant incorporation • Molten‐salt CVD extends to 47+ TMC compositions (32 binary + alloys) • Most widely studied route for device‐quality 2D TMC films	• High temperatures (600–1000 °C); substrate compatibility constraints • Toxic/corrosive precursor gases (H_2_S, H_2_Se) • Expensive quartz tube furnace systems • Batch‐to‐batch reproducibility remains challenging	MediumMonolayer‐few‐layer films; substrate limited	[244, 245, 246, 247]
Hot‐plate/thermal annealing (postdeposition sulphurization/selenization of metal or metal oxide precursor films).	• Simple equipment‐hot plate or tube furnace • Controllable TMC film thickness via precursor film thickness • Compatible with sputtered, evaporated, or solution‐cast precursor films • Mild conditions possible with reactive organochalcogenide precursors (300 °C)	• Toxic chalcogen vapors (S, Se, Te) require specialist gas handling • Phase purity sensitive to temperature and ramp rate • Organic residues can remain when using thiourea/selenourea • Limited to polycrystalline or amorphous thin‐film products	MediumThin films; polycrystalline; wafer‐compatible	[176, 248, 249, 250, 251]
One‐pot heat‐up (all precursors combined from room temperature; slow ramp to reaction temperature‐no injection).		• Simplest colloidal route‐no rapid injection step, safer operation • Scalable to multigramme batches (>30 g demonstrated) • Lower temperatures feasible with reactive ammonium chalcogenide precursors (<200 °C) • Good size and phase control via temperature ramp rate and precursor ratio	HighNanocrystals/QDs; multigramme scale	[83, 176, 250, 252, 253]
Electrospinning (TMC precursor + PAN polymer → nanofibers → carbonization/annealing to TMC@CNF composites) Fiber‐based	• Produces continuous 1D nanofibers with high aspect ratio and large surface area • Easy fiber diameter tuning via voltage, concentration, and viscosity • Seamlessly integrates TMCs with carbon matrix for improved conductivity • Flexible freestanding electrodes achievable—ideal for flexible energy devices	• Requires postelectrospinning annealing/carbonization step (additional process) • Precise control of TMC loading and crystallinity within fibers is complex • Solvent and solution parameter optimization is substrate‐specific • Agglomeration of chalcogenide nanoparticles inside fibers at high loading	Medium1D nanofibers; mat‐scale; flexible electrodes	[141, 252, 254, 255, 256, 257]
Hydrothermal/solvothermal (sealed autoclave; aqueous = hydrothermal; nonaqueous = solvothermal; 120–250 °C, autogenous pressure) Solution‐phase	• Excellent morphology control: nanosheets, nanoflowers, nanotubes, hollow spheres • Wide composition range accessible with simple precursors • Scalable and cost‐effective with standard lab autoclaves • Low reaction temperatures (120–250 °C) compared to solid‐state and CVD	• Opaque autoclave prevents real‐time monitoring of reaction progress • Surfactant/ligand residues on product surfaces impede charge transport • High‐pressure safety requirements for sealed autoclaves • Postsynthesis washing and drying steps needed; crystal quality inferior to CVD	HighNanostructures; broad morphology palette	[176, 258, 259, 260, 261]

Abbreviations: CNF, Carbon nanofibre; PAN, Polyacrylonitrile; QD, Quantum dots.

## 4 Architectural Engineering of Electrocatalysts for Reduction of Nitrate to Ammonia

The electrocatalytic nitrate reduction reaction (NO_3_RR) presents a compelling dual‐function solution: it converts a pervasive pollutant into high‐value ammonia (NH_3_) under ambient temperature and pressure, driven by renewable electricity. This makes NO_3_RR not merely a water‐treatment strategy but a cornerstone of a circular nitrogen economy. A critical distinction must be drawn at first. Much early electrocatalytic ammonia literature reported synthesis via dinitrogen reduction (N_2_RR), where N_2_ from air or dissolved gas was posited as the nitrogen source. Mounting scrutiny has largely invalidated those claims; the N≡N triple bond (941 kJ mol^−1^) is kinetically almost impossible to break electrochemically under mild conditions, and reported yields were frequently contaminated ammonia from the atmosphere, electrolyte, or Nafion membranes. NO_3_RR, by contrast, operates from a far more reactive nitrogen precursor with well‐established, experimentally verified intermediates and offers Faradaic efficiencies exceeding 90% under optimized conditions. The established standard reduction potential for NO_3_RR to NH_3_ is NO_3_^−^ + 9H^+^ + 8e^−^ → NH_3_ + ·3H_2_O, E° = +0.88 V versus reduced hydrogen potential (RHE) [262, 263, 264, 265].

It is known that nitrate's N—O bonds (with bond energy *ca*. 201 kJ/mol) are far weaker and more polarizable than N≡N, meaning adsorption and activation on metal surfaces are energetically accessible. Despite this thermodynamic advantage, the 9‐proton, 8‐electron cascade involved in NO_3_RR generates multiple intermediates (NO_2_^−^, NO, N_2_O, NH_2_OH, NH_2_, etc*.*), each requiring selective stabilization. This multielectronic complexity is the central design challenge. NO_3_RR proceeds through two broadly classified pathways; namely direct and indirect, which differ in the role played by adsorbed hydrogen (H*). In direct pathways, the nitrate ion adsorbs onto the catalyst surface and undergoes stepwise electron and proton transfers without significant involvement of surface H*. In indirect (H‐mediated) pathways*, water is first dissociated to generate adsorbed hydrogen (H* = H^+^ + e^−^), which then chemically reduces adsorbed NO_3_* and its derivatives. Both pathways converge at common intermediates, but the branching point determines selectivity and rate‐determining steps. The consensus mechanistic framework, supported by density functional theory (DFT) computations and isotope labeling, proceeds as follows for the direct pathway [265, 266, 267]:




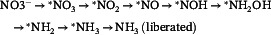




Each arrow represents a proton‐coupled electron transfer (PCET) step.

It is noteworthy to mention that the cogent features of each intermediate are lucidly shown as [268];

1. NO_3_ *^−^* *adsorption and first deoxygenation* (*NO_3_ → *NO_2_): This initial 2‐electron step is often the rate‐determining step (RDS) on many metal surfaces. Strong oxophilicity is advantageous here, and Cu(I) species have been shown by Operando XAS to be the dominant active form during this step [269].
2. *Second deoxygenation* (*NO_2_ → *NO): Requires a second 2‐electron transfer. This step is where N_2_O can form as a by‐product via dimerization of two *NO species if the catalyst binds *NO too weakly, a critical selectivity branch‐point.
3. *Hydrogenation sequence* (*NO → *NOH → *NH_2_OH → *NH_2_ → *NH_3_): These 4‐electron and 5‐proton steps constitute the second half of the cascade. The *NH_2_OH (hydroxylamine) intermediate is a crucial and experimentally detectable node. Conversion of *NH_2_OH → *NH_2_ is frequently the rate‐determining step in total ammonia yield (evidenced by the work of Wu et al. [264] showed tandem systems, where an energy barrier of 0.151 eV was measured at this step).
4. *Alternative branching to* N_2_O *or* N_2_: If two adjacent *NO intermediates couple before further hydrogenation, the N–N dimer pathway produces N_2_O or N_2_, both thermodynamically stable but unwanted by‐products. This is suppressed by catalysts that favor isolated *NO adsorption geometries* (*e.g.*, *single‐atom sites*) *or high H* supply that rapidly intercepts *NO [268].

*In silico* work identified four geometric pathways through the *NO hydrogenation stage, distinguished by which atom (N or O) and which face (end or side) is protonated first at [270];

1. O‐end pathway: H^+^ attacks the oxygen end of *NO → *NOH → continued N—H bond formation.
2. O‐side pathway: Proton attacks the side of the O atom.
3. N‐end pathway: H^+^ attacks the nitrogen end → *HNO.
4. N‐side pathway: Proton attacks lateral to N.

The choice of pathway that predominates depends on the adsorption geometry of *NO on the catalyst surface‐whether it adsorbs N‐down (on late transition metals like Cu, Fe) or O‐down (rare but observed on certain oxides). DFT calculations on Cu(111) and Cu(100) consistently show the N‐end and N‐side pathways to be lower in energy, favoring N—H bond formation and ultimately NH_3_. Authentic NO_3_RR is most rigorously confirmed by ^15^N isotope labeling combined with ^1^H NMR spectroscopy. When ^15^NO_3_^−^ is used as the nitrogen source, the produced ^15^NH_4_^+^ (from ^15^NH_3_ in acidified solution) shows a characteristic doublet NMR pattern at *δ* = 6.80 and 7.04 ppm, distinct from the triplet pattern of ^14^NH_4_^+^ at *δ* = 6.75, 6.93, and 7.10 ppm. This isotope fingerprint unambiguously assigns ammonia production to electrochemical reduction of the added nitrate, not from atmospheric N_2_, dissolved N_2_, or contaminant NH_3_ in reagents. Operando studies further use differential electrochemical mass spectrometry, confirming gaseous NO (m/*z* = 30), NH_3_ (m/*z* = 17), N_2_O (m/*z* = 44), and the critical HNO (m/*z* = 31) and NH_2_OH (m/*z* = 33) intermediates in real time during electrolysis. This rigorous verification protocol sharply distinguishes authentic NO_3_RR from the problematic N_2_RR literature, where ^15^N_2_ gas contamination with trace ^15^NH_3_ and ^15^NO_3_^−^ impurities (as documented in commercial ^15^N_2_ cylinders) produced spurious positive results [270]. It should be well noted that NO_3_RR competes with two principal side reactions, which are (i) HER: 2H^+^ + 2e^−^ → H_2_, thermodynamically favorable and kinetically fast at increasing overpotentials. HER consumes electrons nonproductively and suppresses NH_3_ Faradaic efficiency (FE) and (ii) incomplete reduction to NO_2_^−^, which is observed at low overpotentials or on weakly active catalysts, the reaction stalls at the nitrite stage, giving high NO_2_^−^ selectivity but low NH_3_ yield. Catalyst engineering must therefore simultaneously optimize strong NO_3_^−^ adsorption to prevent HER competition, rapid deoxygenation kinetics, adequate H* supply for the hydrogenation steps and appropriate intermediate binding strengths to prevent accumulation of toxic intermediates [270]. The crystal facet exposed to the electrolyte profoundly influences which reaction pathway operates and what FE is achieved. On copper‐the archetypical NO_3_RR catalyst‐Cu(100) facets dramatically outperform Cu(111). DFT calculations show that Cu(100) binds *NO_3_ more strongly (lower activation barrier for the RDS) due to more undercoordinated surface atoms and a better geometric match between the tetrahedral NO_3_^−^ and the square‐symmetric Cu(100) surface. Studies on electropolished Cu with a predominant (100) surface achieved 91.5 ± 3.7% FE for NH_3_ at −0.4 V versus RHE, compared to significantly lower FE on native oxide‐covered or randomly faceted Cu. Morphology engineering further amplifies active surface area. Nanostructures such as nanowires, nanocubes, nanosheets, and hierarchical foams expose more edge/corner atoms, which are inherently more reactive. Cu_2_O nanocubes offer a particularly illustrative case: at mild cathodic potentials, Cu(I) species in the nanocubes selectively reduce NO_3_^−^ to NO_2_^−^ (FE up to 75%), while at more negative potentials, in situ reduction of Cu(I) to Cu(0) shifts selectivity toward full 8‐electron reduction to NH_3_. Despite the remarkable progress achieved, several challenges still loom, which are scaling current density to industrial levels (>100 mA cm^−2^) while maintaining high FE and preventing electrode deactivation, Operando mechanistic clarity at high current for which most in situ studies are performed at low current densities; intermediate evolution at industrially relevant conditions remains incompletely mapped. Also, long‐term catalyst stability: Cu‐based TMCs reconstruction under reductive conditions, which can be beneficial (as in Cu_2_S → Cu^0^ transition) or detrimental (active site aggregation, leaching) and techno‐economic viability that covers life cycle assessments show NO_3_RR‐to‐NH_3_ can be competitive with Haber–Bosch only when powered by low‐cost renewable electricity and operated at high current densities with high FE simultaneously demanding conditions [270, 271].

## 5 Fundamental Principles of the Electrochemical Nitrate Reduction and Ammonia Production

The fundamental chemical mechanism of NO_3_RR in aqueous media is PCET, which is the simultaneous or sequential transfer of one or more protons and electrons. PCET is the engine of nearly all multielectron redox transformations in artificial electrocatalysis, from the oxygen–evolving complex in photosystem II to NO_3_RR. In PCET, the interdependence of proton and electron transfer introduces mechanistic richness in two extremes, namely stepwise proton transfer–electron transfer, in which proton transfers first to give a protonated intermediate, which then undergoes electron transfer (or vice versa, ET–PT) and *Concerted PCET* (*CPET*) involving the occurrence of proton and electron transfer occur in a single kinetic step, avoiding high‐energy charged intermediates. CPET is thermodynamically more favorable when protonated or deprotonated intermediates would be highly unstable.

In NO_3_RR under aqueous electrochemical conditions, each hydrogenation step of the adsorbed intermediates (*NO_2_, *NO, *NOH, *NH_2_OH, and *NH_2_) proceeds via PCET. Whether each step is concerted or stepwise depends on the relative pK_a_ values of the intermediates and the electrode potential. Electroreduction of nitrate in aqueous systems operates through two mechanistic channels, distinguished by the identity of the hydrogen transfer agent via*:*

1. Direct H^+^/e^−^ mediation: The adsorbed nitrate and its derivatives are directly hydrogenated by protons from the solution and electrons from the electrode, step by step. This is the canonical PCET pathway. The rate depends on both the local proton activity (pH) and the applied electrode potential.
2. Adsorbed H (atomic hydrogen) mediation: Water is first dissociated at the electrode surface (H_2_O + e^−^ → H* + OH^−^ in alkaline media; H^+^ + e^−^ → H* in acidic media) to produce adsorbed atomic hydrogen (H*). H* then reduces the adsorbed nitrogen intermediates chemically rather than electrochemically. Critically, when the hydrogenation is mediated by H*, the formation of N—H bonds is thermodynamically and kinetically more favorable than the formation of N—N bonds. Therefore, H*‐mediated reduction of nitrate is specifically associated with selective NH_3_ production, while H^+^/e^−^ direct reduction is more likely to form N_2_. This distinction has direct design implications: catalysts that efficiently generate and supply H* (e.g., via ruthenium, cobalt, or iron sites with low H* binding energy) favor NH_3_ over N_2_.

The electrode–electrolyte interface, which is the electrical double layer (EDL), has profound consequences for NO_3_RR. These are; (i) Nitrate adsorption: NO_3_^−^ must migrate from the bulk solution through the diffuse layer to the electrode surface, a process governed by electrostatic gradients and the electric field across the EDL. The field at cathodic potentials (negative electrode) repels anions, potentially limiting NO_3_^−^ transport to the surface. Catalyst modifications that preconcentrate NO_3_^−^ at the inner Helmholtz plane (via built‐in electric fields, surface‐positive charges, or specific coordination sites) are therefore highly beneficial and (ii) Local pH effects: During NO_3_RR, proton consumption near the electrode surface creates an alkaline boundary layer even when bulk electrolyte is acidic. This local pH elevation can shift selectivity, favor NH_3_ over N_2_ (by altering PCET equilibria), or cause precipitation of metal hydroxides on the catalyst surface. The electrochemical driving force for any electrode reaction is quantified by the standard reduction potential E°, referenced conventionally to the reversible hydrogen electrode (RHE). The overall NO_3_RR to ammonia is NO_3_^−^ + 9H^+^ + 8e^−^ → NH_3_ + ·3H_2_O E° = +0.88 V versus RHE (at pH 0). Thermodynamically, this is more favorable than molecular nitrogen reduction, which is N_2_ + 6H^+^ + 6e^−^ → 2NH_3_ E° = +0.55 V versus RHE (at pH 0). The 0.33 V of NO_3_RR combines with the far lower N—O bond energy (204 kJ/mol) versus N≡N (941 kJ/mol), explains why NO_3_RR achieves practical FE exceeding 90% under mild conditions while N_2_RR remains largely impractical at the electrochemical scale [65, 78, 272, 273].

Next decade research on TMC electrocatalysts will depend on the synergistic integration of theory‐guided material design, realistic performance evaluation, and advanced Operando characterization. DFT‐assisted discovery of novel TMCs can accelerate the identification of highly active and selective catalysts, while comprehensive anti‐poisoning studies in authentic wastewater environments will bridge the gap between laboratory research and practical application. Meanwhile, in situ and Operando characterization techniques will provide unprecedented insights into the dynamic evolution of active sites and reaction mechanisms. Collectively, these research directions are expected to drive the development of robust, efficient, and scalable TMC electrocatalysts for sustainable nitrate remediation and ammonia production [247, 274, 275].

## 6 Applications of TMCs for Nitrate Reduction

The applications of TMCs, e.g., MoS_2_, WS_2_, MoSe_2_, FeS_2_, CoSe_2_, NiS_2_) for nitrate reduction are tied to their attractiveness with respect to their tunable electronic structure (phase engineering 2H *vs* 1T, d‐band tuning) that enables the controlled adsorption/activation of nitrate and intermediates, rich defect chemistry (chalcogen vacancies, edges) that provides active sites; good conductivity (especially metallic phases like 1T‐MoS_2_, certain selenides) for electrocatalysis; structural versatility: nanosheets, nanoparticles, nanoflowers, and porous frameworks for high surface area and compatibility with single‐atom/cluster dispersion and heterostructuring (with carbons, oxides, and metals) to improve activity/selectivity. TMCs are used as [247, 274, 275, 276];

1. Promising electrocatalysts for nitrate reduction due to their strong potential for nitrogen resource recovery and renewable‐energy‐driven electrosynthesis. Suppression of hydrogen evolution reaction (HER) while promoting ammonia formation.
2. Cathode catalysts for electrochemical nitrate reduction to NH_3_, N_2_, or NO_2_^−^.
3. Photo(electro)catalysts with suitable bandgaps harvest light to drive photo‐assisted nitrate reduction (photocatalytic or photoelectrochemical), often combined with cocatalysts.
4. Heterogeneous thermal catalysts, such that TMCs (sulfides/selenides) can serve as supports in thermocatalytic nitrate hydrogenation.
5. Decentralized water remediation: electrodes based on TMCs for electrocatalytic removal/reduction of nitrate in drinking/groundwater, with option to recover NH_3_. Portable electrochemical nitrate reduction units use TMC electrodes for groundwater treatment in remote agricultural regions and rural communities.
6. Sustainable NH_3_ production: electrosynthesis of ammonia from nitrate feedstocks (wastewater‐derived nitrate) at low overpotentials.
7. Photoelectrochemical reactors coupling TMC photocathodes with oxidizing photoanodes for solar‐driven nitrate‐to‐ammonia conversion.

A critical comparison of the recent literature indicates that the outstanding performance reported for TMC‐based materials often results from sophisticated nanostructure engineering, defect creation, heterostructure formation, or hybridization with highly conductive materials. While these strategies significantly enhance material performance, they frequently increase synthesis complexity, production cost, and challenges in reproducibility. Furthermore, direct comparison among published studies is hindered by the lack of standardized testing protocols, inconsistent reporting of experimental conditions, and variations in material composition and morphology. Another notable limitation is that many studies emphasize achieving record performance metrics while providing limited discussion of scalability, environmental sustainability, manufacturing costs, and long‐term operational stability. Future research should therefore shift from maximizing laboratory‐scale performance toward developing scalable synthesis methods, standardized characterization procedures, realistic device testing, mechanistic understanding of degradation processes, and comprehensive techno‐economic and life‐cycle assessments. Such efforts will enable more meaningful comparisons among studies and accelerate the commercialization of TMC‐based technologies [247, 274, 275, 276, 277, 278, 279, 280].

Over the years, researchers have utilized the various electrocatalysts for the conversion of nitrate to ammonia.

Zhang et al. [281] utilized MoS_2_ electrocatalyst for the reduction of nitrate to ammonia and obtained a FE of 1.17% for ammonia synthesis at −0.5 V versus RHE in 0.1 M Na_2_SO_4_.

Ding et al. [282] worked on iron‐doped MoS_2_ nanosheets and achieved a maximum of 90% FE for ammonia synthesis at −0.85 V versus RHE in aqueous electrolyte.

Also, Li et al. [283] used defect‐rich MoS_2_ nanoflowers for the electrochemical synthesis of ammonia at 8.34% FE at −0.40 V versus RHE in 0.1 M Na_2_SO_4_. This study showed that at −0.85 V versus RHE at −0.40 V versus RHE in 0.1 M Na_2_SO_4_, the defect‐free counterpart of MoS_2_ nanoflowers achieved a maximum of 2.18% FE and ammonia yield of 13.41 μg h^−1^ mg^−1^, which was low when compared to that of defect‐rich MoS_2_ nanoflowers (29.28 μg h^−1^ mg^−1^).

Sun et al. [284] obtained ammonia from the simultaneous multielectron reduction of nitrogen, which decreased the energy barrier for nitrogen photo‐reduction in this case. Infact, this study showed that the concentration of ammonia changed with respect to various synthesis techniques, type and pH. For instance, ammonia yield decreased in the order sonication at acidic pH > hydrothermal > sonication at neutral pH > commercial ultrathin MoS_2_ nanosheets.

Suryanto et al. [285] achieved a maximum of 17.6% FE for ammonia synthesis at −150 mV versus RHE in 10 mM HCl after 4 h and the polymorphic engineering of Ru/MoS_2_ improved its selectivity for nitrate conversion to ammonia by suppressing H^+^ reduction to gaseous hydrogen.

For Dong et al. [286], the engineering of crystal phase by the twinning of Zn_0.8_Cd_0.2_S nanocrystals revealed an increase in the excitation of electrons, direct transport of charge carriers, efficient electron supply, and appropriate tuning of energy bandgap structure. Eventually, this led to an increase in ammonia yield in 1 mM Na_2_SO_3_. The report from this study revealed that NH_3_ production rate of 0.14–3.94 μg h^−1^ mg^−1^, within an apparent quantum efficiency (AQE) of 0.14–2.53 for 2 h in the presence of visible light (VL) irradiation in Na_2_SO_3_ (sacrificial agent).

Ye et al. [287] demonstrated that maximum ammonia yield was achieved at AQE of 0.043 after 1 h of VL irradiation.

Shen et al. [288] and Sun et al. [289] showed that metal‐free cocatalysts (black phosphorus crystal nanosheet (BPCN) and V‐1T‐MoS_2_/Pt) enhanced the efficiency in ammonia yield.

The tuning of electrocatalysts to produce new active sites improves the adsorption of nitrogen, dissociation of nonpolar N≡N bond, and in turn increases the electrochemical yield of ammonia, according to Hu and his Colleagues [290].

Qin and his coresearchers [291] investigated that doping Mo_1−*x*_W_*x*_S_2_ (*x* = 0.32) with W increased electron cloud density of its 5d orbital, which resulted in the polarizability and activation of nitrogen molecules available for adsorption. Thus, this increased the synthesis yield of ammonia. Surface defect engineering via the introduction of oxygen vacancies into TiO_2_‐doped WS, sulfur vacancies into In_2_S_3_ and Zn vacancies into Zn_3_In_2_S_6_ by Shi et al. [292], He et al. [293] and Han et al. [294] respectively, revealed that charge separation efficiency was enhanced, shelf life of charge carriers was prolonged, and the electrochemical ammonia yield was improved as well. Swain et al. [295] explored the creation of S−S links from p‐MoS_2_/n‐MgIn_2_S_4_ heterojunctions produced abundant exposed edge sites that were responsible for the effective photogeneration of charge carriers. Lu [296] et al. investigated that very low specific surface area (∼2 m^2^.g^−1^) Fe_2_Mo_6_S_8_ displayed intrinsic behavior in aqueous electrolytes for the electrochemical reduction of nitrogen to ammonia with 12.5% FE at −0.20 V versus RHE and specific electrocatalyst surface area‐normalized rate of 70 μg h^−1^ mg^−1^. Fu et al. [297] observed that the structured inclusion of Mn sites into Re_2_MnS_6_ resulted in the emergence of nonbonding semimetal states and structural phase transition that lucidly reduced the activation energy barrier of the reaction coordinate for the electrochemical production of ammonia.

Li et al. [298] reported that Co_2_Mo_6_S_8_ gave a biocatalyst‐like appreciable turnover frequency of ∼95.1 s^−1^, 97.1% FE at −0.41 V versus RHE in 0.1 M NaCl for the electrochemical production of ammonia.

Xia et al. [299] reported that anions with smaller electronegativity and larger size in Ni_2_Mo_6_S_8_, Ni_2_Mo_6_Se_8_, and Ni_2_Mo_6_Te_8_ elicit high‐level inhibition of competing hydrogen evolution reaction and direct hydrogenation for selective ammonia production, FE selectivity up to 99.4% and turnover frequency up to 21.5 s^−1^.

Yang et al. [300] reported that chemical interaction between CoS_2_ and MoS_2_ modulated interfacial electronic charge transfer and charge distribution between both electrocatalysts, which elicited the emergence of local electrophilic sphere very close to CoS_2_, for efficient absorption of nitrogen, as well as the emergence of nucleophilic sphere very close to MoS_2_ for the disintegration of stable N≡N and the decrease of activation energy barrier via potential‐determining step (*N_2_ → *N_2_H).

It is interesting to note here that Wang et al. [301] synthesized rare‐earth La‐doped VS_2‐x_ for the electrochemical reduction of nitrate to ammonia. Wang and his research team observed that ammonia was obtained at 96.6% FE at −0.60 V versus RHE. Also, their report revealed that theoretical computations using La‐dopants and sulfur vacancies mutualistically promoted nitrate activation, activation energy barrier reduction, and hydrogen evolution reduction. Guo et al. [302] reported that 18.1% FE at −0.84 V versus RHE was achieved for the production of ammonia by FeTe_2_.

Also, Wang and his coresearchers reported that the electrochemical conversion of nitrogen to ammonia by M–Te (M = Ru, Rh, Ir) was achieved at 15.3% FE, with a shelve‐life of 20 consecutive cycles [303].

It is important to understand that the reduction of nitrate and oxidation of water take place at the cathode and anode respectively, as depicted in the following reduction–oxidation process Equations (I) to (V) [304];

(I) $$3H_{2}O\to 3/2O_{2}+9H+6e^{-}\left(\text{Anode};\;\text{acidic}\right)$$

(II) $$6H+6e^{-}+N_{2}\to 2{\mathrm{NH}}_{3}\;\left(\text{Cathode};\;\text{acidic}\right)$$

(III) $$6{\mathrm{OH}}^{-}\to 3/2O_{2}+3H_{2}O+6e^{-}\left(\text{Anode};\;\text{alkaline}\right)$$

(IV) $$6H_{2}O+N_{2}+6e^{-}\to 2{\mathrm{NH}}_{3}+6{\mathrm{OH}}^{-}\left(\text{Cathode};\;\text{alkaline}\right)$$

(V) $$N_{2}+3H_{2}O\to 3/2O_{2}+2{\mathrm{NH}}_{3}\;\left(\text{Overall}\;\text{REDOX}\right)$$

At ambient conditions, electrochemical reduction of nitrate to ammonia is understood to have a promising potential of not increasing the carbon footprint in the environment, hence improving environmental sustainability and ecological balance. The ubiquitous nature of nitrogen and water (carbon‐free reactants) opens up new possibilities for the utilization and conversion of mammoth nitrogen in the atmosphere to ammonia (Equations (VI) to (XIII) by efficient electrocatalysts [305].

**FIGURE 4:**
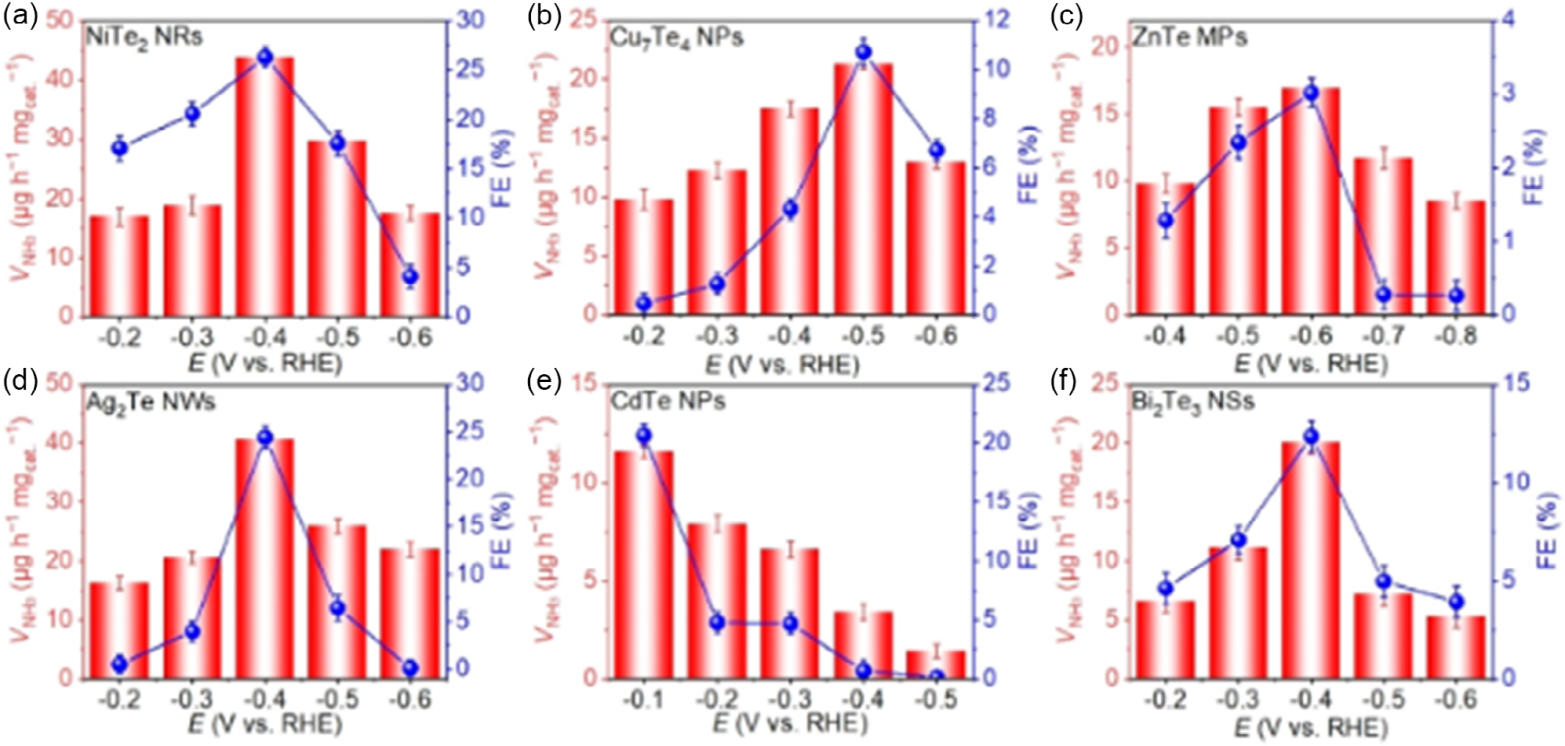
Ammonia yield rates for (a) NiTe_2_, (b) Cu_7_Te_4_, (c) ZnTe, (d) Ag_2_Te, (e) CdTe, and (f) Bi_2_Te_3_ [307]. Reproduced with permission: Copyright 2024, Elsevier.

**FIGURE 5:**
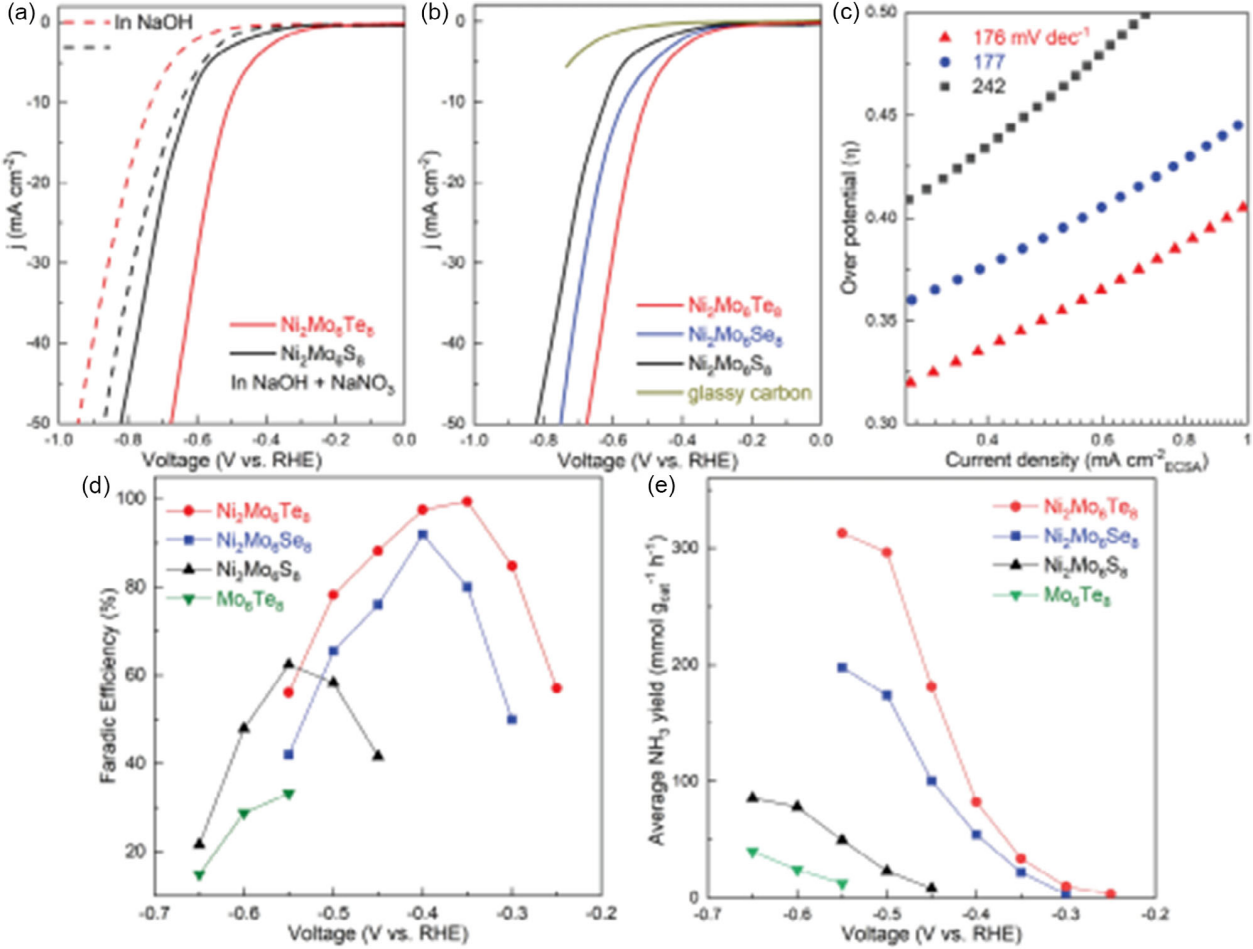
Electrocatalytic performance of Ni_2_Mo_6_Te_8_ for nitrate reduction to ammonia: comparison of linear sweep voltammogram (LSV) at 5.0 mV s^−1^ between (a) Ni_2_Mo_6_Te_8_ and Ni_2_Mo_6_S_8_ in 0.5 M NaOH with and without 0.5 M NaNO_3_; (b) LSV curves; (c) corresponding Tafel plots of nitrate reduction kinetics on glassy carbon electrodes modified with different types of catalysts in 0.5 M (NaOH+NaNO_3_); (d) Faradic efficiency; and (e) ammonia yield with respect to catalyst over‐potentials [299]. Reproduced with permission: Copyright 2024, Wiley‐VCH GmbH.

**FIGURE 6:**
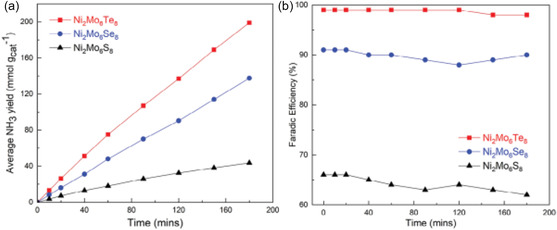
Electrocatalyst durability test for; (a) average yield rate of ammonia and (b) Faradic efficiency for 3 h [299]. Reproduced with permission: Copyright 2024, Wiley‐VCH GmbH.

Table 2 shows the rate of yield of ammonia by NiTe_2_, Cu_7_Te_4_, ZnTe, Ag_2_Te, CdTe, and Bi_2_Te_3_. Figures 4, 5, 6 show the electrochemical performance of various TMCs for the conversion of nitrate to ammonia [309].

**TABLE 2:** Various TMCs and their ammonia yield.

TMC	NH_3_ yield	Electrolyte composition and concentration	Applied potential versus RHE	% FE	References
Defect‐rich MoS_2_ nanoflowers	29.28 μg h^−1^ mg^−1^	0.1 M Na_2_SO_4_	−0.85 V	8.34	[283]
Ultrathin MoS_2_ nanosheets	19.12 μg h^−1^ mg^−1^	0.5 M Na_2_SO_4_	−0.24 V	—	[284]
Zn_0.8_Cd_0.2_S	3.94 μg h^−1^ mg^−1^	0.1 M Na_2_SO_3_	—	—	[286]
Ni_2_P/Zn_0.5_Cd_0.5_S	14.94 μg h^−1^ mg^−1^	0.1 M Na_2_SO_4_	—	—	[287]
BPCN/CdS	14.13 μg h^−1^ mg^−1^	0.5 M Na_2_SO_4_	−0.05 V	—	[288]
O‐doped V‐1T‐MoS_2_/CdS	0.48 μg h^−1^ mg^−1^	0.5 M Na_2_SO_4_	—	—	[289]
Mn‐doped MoS_2*−x*_	12.54 μg h^−1^ mg^−1^	0.5 M Na_2_SO_4_	—	—	[290]
Mo_0.68_W_0.32_S_2_	6.53 μg h^−1^ mg^−1^	0.1 M Na_2_SO_3_	−0.5 V	—	[291]
TiO_2_ doped WS	0.08 μg h^−1^ mg^−1^	0.5 M Na_2_SO_4_	—	—	[292]
In_2_S_3_ nanotubes	3.09 μg h^−1^ mg^−1^	0.5 M Na_2_SO_4_	—	—	[293]
Fe_2_Mo_6_S_8_	70 μg h^−1^ mg^−1^	0.5 M Na_2_SO_4_	−0.20 V	12.5	[296]
Re_2_MnS_6_	3.78 μg h^−1^ mg^−1^	0.1 M Na_2_SO_4_	−0.3 V	17.42	[297]
Co_2_Mo_6_S_8_	6.79 μg h^−1^ mg^−1^	0.17 M NaSO_4_ + 0.5 M NaNO_3_	−0.67 V	97.1	[298]
Ni_2_Mo_6_Se_8_	11.59 μg h^−1^ mg^−1^	0.5 M NaOH + 0.5 M NaNO_3_	−0.40 V	99.4	[299]
Ni_2_Mo_6_S_8_	2.88 μg h^−1^ mg^−1^	0.5 M NaOH + 0.5 M NaNO_3_	−0.38 V	62	[299]
Ni_2_Mo_6_Te_8_	18.41 μg h^−1^ mg^−1^	0.5 M NaOH + 0.5 M NaNO_3_	−0.25 V	57	[299]
CoS_2_/MoS_2_	54.7 μg h^−1^ mg^−1^	1.0 M NaOH	−0.6 V	20.8	[300]
Cu–SnS_2−*x*_	0.04 μg h^−1^ mg^−1^	0.1 M KOH + 0.1 M KNO_3_	−0.7 V	93.8	[306]
FeTe_2_	39.2 μg h^−1^ mg^−1^	0.32 M NaOH, 0.4 M C_7_H_6_O_3_Na	−0.84 V	18.1	[302]
IrTe_4_	51.1 μg h^−1^ mg^−1^	0.1 M KOH	−0.20 V	15.3	[303]
NiTe_2_	43.8 μg h^−1^ mg^−1^	0.1 M HCl	−0.4 V	26.3	[307]
Ag_2_Te	40.6 μg h^−1^ mg^−1^	0.1 M HCl	−0.4 V	24.4	[307]
Cu_7_Te_4_	21.3 μg h^−1^ mg^−1^	0.1 M HCl	−0.5 V	10.7	[307]
Bi_2_Te_3_	20.1 μg h^−1^ mg^−1^	0.1 M HCl	−0.4 V	12.4	[307]
ZnTe	16.9 μg h^−1^ mg^−1^	0.1 M HCl	−0.6 V	3.0	[307]
Fe_3_S_4_	75.4 μg h^−1^ mg^−1^	1 M NaOH	−0.4 V	6.45	[308]

(VI) $$N{{O_{3}}^{-}}_{\left(\mathrm{aq}\right)}\to N{{O_{3}}^{-}}_{\left(\mathrm{ad}\right)}$$

(VII) $$H_{2}O+{e^{-}}_{\left(\mathrm{aq}\right)}\to H_{\left(\mathrm{ad}\right)}+{\mathrm{OH}}^{-}$$

(VIII) $$N{{O_{3}}^{-}}_{\left(\mathrm{ad}\right)}+2H_{\left(\mathrm{ad}\right)}\to N{{O_{2}}^{-}}_{\left(\mathrm{ad}\right)}+H_{2}O$$

(IX) $$N{{O_{2}}^{-}}_{\left(\mathrm{ad}\right)}+H_{\left(\mathrm{ad}\right)}\to {\mathrm{NO}}_{\left(\mathrm{ad}\right)}+{\mathrm{OH}}^{-}$$

(X) $${\mathrm{NO}}_{\left(\mathrm{ad}\right)}+2H_{\left(\mathrm{ad}\right)}\to N_{\left(\mathrm{ad}\right)}+H_{2}O$$

(XI) $$N_{\left(\mathrm{ad}\right)}+H_{\left(\mathrm{ad}\right)}\to {\mathrm{NH}}_{\left(\mathrm{ad}\right)}$$

(XII) $${\mathrm{NH}}_{\left(\mathrm{ad}\right)}+H_{\left(\mathrm{ad}\right)}\to {\mathrm{NH}}_{2\left(\mathrm{ad}\right)}$$

(XIII) $${\mathrm{NH}}_{2(\mathrm{ad})}+H_{\left(\mathrm{ad}\right)}\to {\mathrm{NH}}_{3(\mathrm{ad})}$$

## 7 Conclusion and Future Perspectives

Although TMCs have demonstrated remarkable potential as electrocatalysts for nitrate reduction and other electrochemical environmental applications, several critical challenges remain that limit their practical implementation. Future research should focus on the following:

1. The development of next‐generation TMC electrocatalysts should increasingly rely on DFT calculations to establish structure–activity relationships and accelerate catalyst discovery. Conventional trial‐and‐error synthesis is both time‐consuming and resource‐intensive, whereas DFT enables the prediction of catalytic activity before experimental validation. Future DFT studies should focus on (i) identifying optimal transition metal combinations in binary, ternary, or high‐entropy chalcogenides that exhibit suitable adsorption energies for key nitrate reduction intermediates (*NO_3_, *NO_2_, *NO, *NH_2_OH, and *NH_3_), (ii) engineering electronic structures through elemental doping, alloying, strain modulation, and vacancy creation to optimize the d‐band center and improve reaction kinetics, (iii) predicting the effects of chalcogen substitution (S, Se, and Te) on conductivity, catalytic activity, and reaction selectivity, (iv) constructing heterostructures and interface‐rich catalysts whose charge redistribution can promote electron transfer and stabilize reaction intermediates, and (v) employing machine learning integrated with DFT databases to rapidly screen thousands of candidate materials and identify promising catalysts with high activity, selectivity, and stability.
2. Development of catalysts with enhanced anti‐poisoning capability for real wastewater applications. Electrocatalytic performances are evaluated using synthetic electrolyte solutions containing only nitrate ions. However, actual industrial, municipal, and agricultural wastewaters contain numerous coexisting contaminants that can significantly deteriorate catalyst performance. Future studies should systematically investigate catalyst resistance against poisoning by species such as chloride, sulfate, phosphate, carbonate, bicarbonate, silicates, natural organic matter, suspended solids, and biofilms. These species may compete for active sites, alter adsorption behavior, induce catalyst corrosion, or promote undesirable side reactions, thereby reducing catalytic efficiency and long‐term durability. Also, future catalyst design should emphasize (i) surface engineering strategies that suppress competitive adsorption while maintaining high nitrate affinity, (i) protective yet conductive coatings that prevent surface deactivation, and (iii) defect engineering that preserves active sites under harsh operating conditions. Long‐term continuous‐flow experiments using real wastewater samples should become standard practice. Performance metrics should include catalyst lifetime, regeneration capability, structural stability, FE, energy consumption, resistance to contaminant accumulation, and ammonia selectivity over extended operational periods. These evaluations will provide a more realistic assessment of catalyst performance and commercial feasibility.
3. Investigation of electrochemical surface reconstruction. Increasing evidence suggests that the active catalytic phase is often generated in situ during operation through oxidation, reduction, chalcogen leaching, vacancy formation, or phase transformation. Future headway in this research should focus on the likelihood of original TMCs remaining catalytically active or serves merely as a precursor to the true active phase.
4. Investigation of electrochemical surface reconstruction. Increasing evidence suggests that the active catalytic phase is often generated in situ during operation through oxidation, reduction, chalcogen leaching, vacancy formation, or phase transformation. Understanding these dynamic processes will enable the intentional design of catalysts that reconstruct into highly active and stable phases during operation.
5. Beyond achieving high catalytic activity in laboratory‐scale experiments, future research should emphasize practical deployment. Significant directions should include (i) scaling up catalyst synthesis using cost‐effective and environmentally sustainable methods, designing durable electrodes suitable for continuous‐flow electrochemical reactors, and (iii) evaluating catalyst performance under industrial current densities and long‐term operation, and (iv) the standardization of testing protocols and benchmarking procedures should also be established to enable meaningful comparisons among different catalyst systems.

## Nomenclature

AQE: apparent quantum efficiency
BPCN: black phosphorus crystal nanosheet
C_B_
: conduction band
CVD: chemical vapor deposition
D: cm
E: V.m^‐1^
EC: European Commission
FDU‐5: Fudan University‐5
FE: Faradaic efficiency
I: AKIT‐6 Korean Institute of Technology‐6
MCM‐48: mobil composition of matter ‐48
Q: mL.h^−1^
R: cm
REDOX: reduction–oxidation
RHE: reversible hydrogen electrode
ROS: reactive oxygen species
SBA‐15: Santa Barbara amorphous‐15
TM: transition metal
TMCs: transition metal chalcogenides
USEPA: United States Environmental Protection Agency
UV: ultra violet
V_B_
: valence band
VL: visible light
WHO: World Health Organisation
ρ: g.cm^‐3^

## Author Contributions

**Martins O. Omorogie**: conceptualization, data curation, supervision, validation, visualization, writing – original draft, writing – review and editing.

## Funding

This study was supported by The Alexander von Humboldt Stiftung, Germany and Carl Friedrich von Siemens Stiftung, Germany.

## Conflicts of Interest

The author declares no conflicts of interest.

## Data Availability

These datasets that support these findings are accessible in the public domain.
